# Acute Digital Ischemia After Arterial Injection of Crushed Zolpidem Tablets: Role of Microcrystalline Cellulose? A Case Report

**DOI:** 10.3389/fphar.2020.560382

**Published:** 2020-12-03

**Authors:** Grégoire Détriché, Guillaume Goudot, Lina Khider, Alexandre Galloula, Matthieu Guillet, Agnès Lillo-Le Louët, Emmanuel Messas, Tristan Mirault

**Affiliations:** ^1^ Vascular Medicine, Hôpital Européen Georges-Pompidou, Assistance Publique Hôpitaux de Paris, APHP centre Université de Paris, Paris, France; ^2^ INSERM U970, Paris Cardiovascular Research Center, Paris, France; ^3^ Physics for Medicine Paris, INSERM U1273, ESPCI Paris, CNRS FRE 2031, PSL Research University, Paris, France; ^4^ Centre Régional de Pharmacovigilance de Paris–Hôpital Européen Georges-Pompidou, Assistance Publique Hôpitaux de Paris, APHP centre Université de Paris, Paris, France; ^5^ VASC European Research Network, Centre de Référence des Maladies Vasculaires Rares, Hôpital Européen Georges-Pompidou, Assistance Publique Hôpitaux de Paris, APHP centre Université de Paris, Paris, France

**Keywords:** case report, microcrystalline cellulose, zolpidem, drug misuse, acute digital ischemia

## Abstract

Literature is scarce on acute ischemia after intra-arterial injection of crushed tablets and no effective medical treatment against the progression of lesions is reported. The only factor able to modify the outcome is the delay between injection and management by a specialized vascular team. Moreover the risk of necrosis seems higher after benzodiazepine intra-arterial injection than with other drugs. We tried to find out mechanistic explanations. We report on the case of a 31-year-old drug addict woman who self-injected into her left brachial artery crushed tablets of zolpidem. She developed an acute ischemia of the left hand, with necrosis of the intermediate and distal phalanges of fingers II, III, and IV. Angiogram of the left upper arm confirmed the distal arterial occlusions with no run-off after the palmar arch in the necrotic fingers. Once she was admitted into our vascular unit, intravenous vasodilator therapy by iloprost, heparin and local protective care were rapidly introduced. After delineation between living and necrotic tissues, she required distal amputations of the affected fingers. The clinical severity of arterial injections of benzodiazepine tablets is linked to the association of several pathophysiological mechanisms. Rather than related benzodiazepine pharmacologic effects with tissue ischemia, by the inhibition of phosphodiesterase, a vasodilator intermediate, or through the peripheral benzodiazepine-type receptor, the predominant mechanism is more likely in relation with microcrystalline cellulose, one component of zolpidem tablets, known as potential embolic agents. They are insoluble and resistant to degradation in water. These properties are probably prominent in the case we described here. Through this case report we want to drag attention of physicians in charge of a patient with acute ischemia after crushed tablet accidental intra-arterial injection, not only to look at the drug injected but also the other components of the tablet and especially to microcrystalline cellulose.

## Introduction

Many reports and reviews have been published on intravenous injection of crushed tablets and their local and systemic pathologic effects especially pulmonary ones (Partanen et al., 2009; Jonsson et al., 2014). The literature is less abundant on acute ischemia after intra-arterial injection of crushed tablets. Pathophysiologic mechanism remains unclear despite many hypotheses have been proposed (Sen et al., 2005).

Moreover no effective medical treatment against the progression of lesions is reported. The only factor able to modify the outcome is the delay between injection and management by a specialized vascular team (Devulapalli et al., 2015). Moreover the risk of necrosis seems higher after benzodiazepine intra-arterial injection than with other drugs. Several mechanisms have been suggested in relation with benzodiazepine pharmacological properties. We report here the case of crushed zolpidem tablet intra-arterial injection and found out that the drug itself may not be the main culprit for digital necrosis whereas the microcrystalline cellulose spheres, one component of the tablet, could.

## Case Description

A 31-year-old female patient was admitted to the vascular medicine department of the Hôpital Européen Georges-Pompidou for an acute ischemia of the left hand. She presented with necrosis of the intermediate and distal phalanges of the second, third, fourth and fifth fingers of her left hand.

Three days earlier, the patient turned up at the primary care center close to her home for a sudden and constant pain in her left hand. She was discharged within 2 h with painkillers. After 72 h, cyanosis and coldness of the intermediate and distal phalanges of the fingers of the left hand appeared. She then decided to consult at the Hôpital Européen Georges-Pompidou emergency department.

**FIGURE 1:**
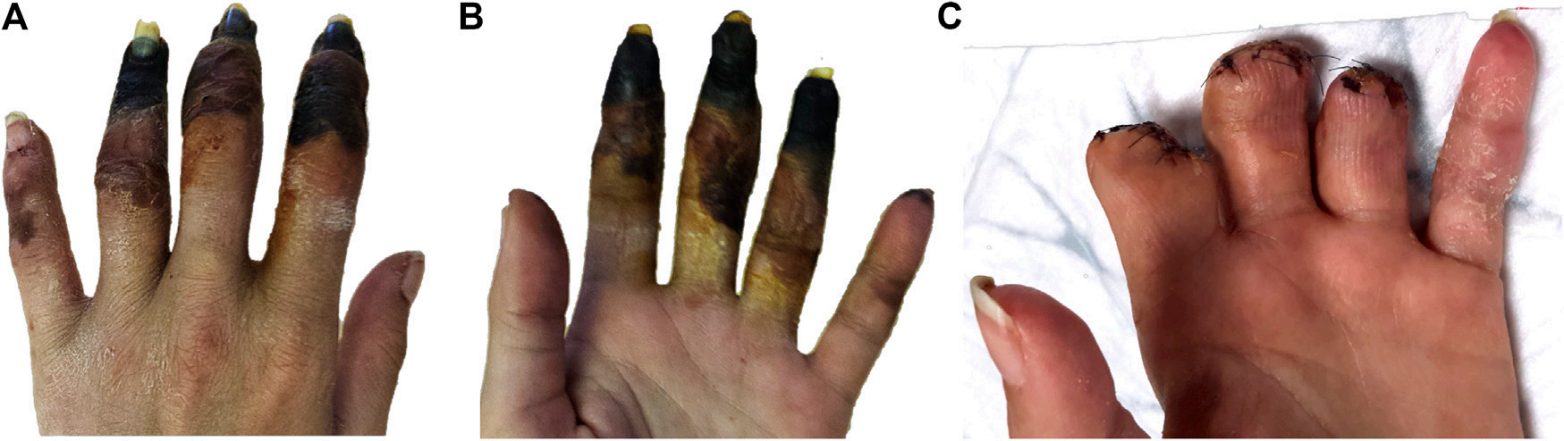
Ischemia of the left hand fingers 72 h after intra-arterial drug injection **(A)** prone position, **(B)** supine position and **(C)** after amputations.

Left hand examination revealed the following injuries: cyanosis and coldness of the intermediate and distal phalanges of the fifth finger, necrosis of the distal phalanges of the second, third and fourth fingers, phlyctens of the intermediate phalanges of the second, third and fourth fingers and the proximal phalanges of the fourth finger (Figures 1A,B). The hand was still painful with paresthesia, but without fingers paralysis. Upper limb pulses were present and symmetrical. Injection marks were noted at the bend of the right and left elbows. Cardiac auscultation was normal without any bruit that could evoke an infective endocarditis, and she had no fever. Electrocardiogram showed a sinus rhythm without any abnormalities.

In her medical history, she had no cardiovascular history but active smoking, as her only cardiovascular risk factor. Spontaneously, she mentioned being a drug addict, but weaned off ectasy, codein, and cannabis, and currently in a weaning off program for heroin. She had been prescribed tapered doses of buprenorphine but still used to take bromazepam and zolpidem off the counter.

**FIGURE 2:**
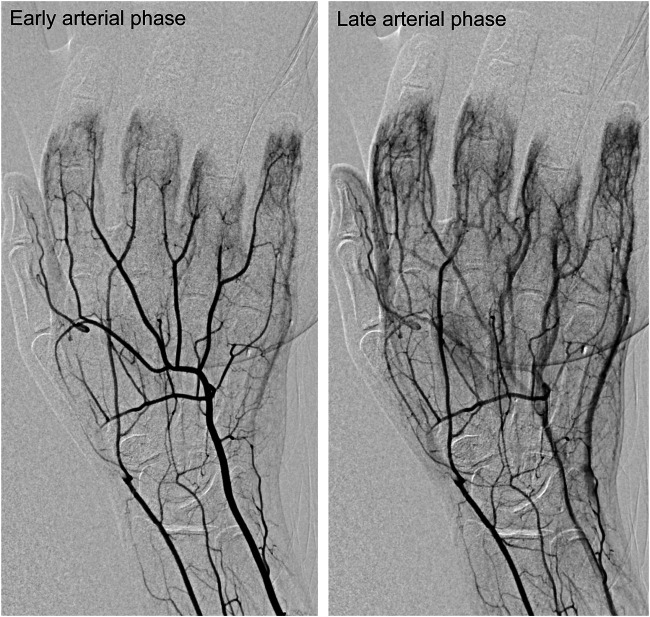
Digital subtracted arteriography of the left hand showing occlusion of the distal portion of the radial artery and of all interdigital arteries of the second, third, fourth and fifth fingers at an early arterial phase **(left panel)** and late arterial phase **(right panel)**.

The continuous Doppler confirmed the presence of flow in the left radial, ulnar and palmar arteries. A digital subtraction angiography was rapidly performed which demonstrated no run-off in the digital arteries of the second, third and fourth left fingers (Figure 2). No thromboaspiration could be performed on these too small arteries. Anticoagulation with unfractionated heparin was started.

The patient was transferred to the vascular medicine department for medical therapy and further investigations. As the patient denied any recent intravenous drug injection, a complete screening for acute limb ischemia etiology was performed.

Human Immunodeficient Virus and Hepatitis B Virus serology were negative, whereas Hepatitis C Virus serology came back positive. Several analyses to find a cryoglobulinemia returned negative.

Arterial thrombophilia has been investigated in vain, with normal prothrombin time and activated partial thromboplastin time, absence of anti-cardiolipin antibodies, anti-beta2GPI antibodies, lupus anticoagulant, antinuclear antibodies, anti-double stranded DNA antibodies, anti polymorphonuclear cytoplasm antibodies. There was no antithrombin or protein S or protein C deficiency and no hyperhomocysteinemia. There was no argument for a myeloproliferative syndrome in the absence of a clinical call point (especially no mass or lymphadenopathy), the blood count was unspecialized and JAK2 mutation V617F was absent.

We also looked for an embolic arterial cause. The *trans*-thoracic echocardiography did not find any thrombus or any argument for an infectious endocarditis. The 24 h Holter-ECG was normal. The computed tomography performed at the emergency room did not show any atheroma on the aortic arch or atheromatous plaque on the left upper limb arteries.

Once she was arrived in our department, we started a daily treatment with iloprost, an arterial vasodilator, as well as pain medication, and local care with a protective dressing. Treatment with iloprost allowed delineation of necrosis at the intermediate and distal phalanges of the three fingers involved, and cyanosis of the fifth finger and the proximal phalanx of the fourth finger resolved. Management by an orthopedic surgical team specialized in the upper limb and hand was required for the amputation of the intermediate and distal phalanges of the three fingers involved (Figure 1C).

Finally, the patient acknowledged she had injected herself, 3 days before her admission, just below the fold of her left elbow with one and a half crushed tablet of zolpidem 10 mg mixed with contact lens cleaning fluid. The patient intended to do an intravenous injection, but it turned out to be an accidental brachial intra-arterial injection. The 3 days delay corresponded to the beginning of the pain and to her first consultation at the local medical center.

**FIGURE 3:**
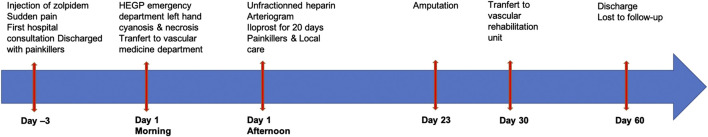
Timeline summary.

Antithrombotic therapy by anticoagulants was changed for antiplatelet therapy and the patient was then transferred to a vascular rehabilitation unit for complete healing of the amputation wounds. Figure 3 presents a timeline summary. After her discharge, the patient did not come back at follow-up consultations and was lost to follow-up despite several reminders.

## Discussion

Intra-arterial injection of crushed tablets of a benzodiazepine-like imidazopyridine derivative is a rare but particularly serious and devastating phenomenon, as evidenced by our observation, which describes a 31-year-old drug-addict patient who misused zolpidem by injecting crushed zolpidem tablets in her brachial artery. With regard to care, we chose to carry out an arterial vasodilator therapy using iloprost in order to limit the digital necrosis as much as possible. In addition to the examination of the patient regarding the misuse of zolpidem, we nevertheless carried out an extensive check-up looking at curable causes and better prognosis etiologies with arterial occlusion or embolization. Very few cases have been described so far (Chang and Lin, 2003; Boucher et al., 2008), other cases being intra-venous injection of zolpidem (Kao et al., 2004; Brunelle et al., 2005; Benyamina et al., 2007; Hsu and Chiu, 2012). In 2008, (Boucher et al., 2008) reported the case of a 35-year-old male who injected a crushed zolpidem tablet into his right forearm and consulted at the emergency room 4 h later. Therapeutic management consisted of anticoagulants associated with antiplatelet therapy and a vasodilator. From the initially ischemic second, third and fourth right fingers, only the last phalanx of the third finger was amputated. In 2003, (Chang and Lin, 2003) reported the case of a 24-year-old female with a crushed zolpidem tablet injected into her right brachial artery. She presented to the emergency room 48 h after. Despite anticoagulation, antibiotic therapy and intravenous vasodilators, the ischemia progressed toward necrosis of the first four fingers of the right hand. After questioning the French national pharmacovigilance database, four other cases of digital necrosis and amputation after intra-arterial injection of crushed tablets were identified: two with crushed buprenorphine tablet, one with amineptine and one with zolpidem. The patients were heroin addicts between 32 and 49 years old. Digital amputation was required for all patients; no information on medical management was described. The available literature on this subject is rare, contrary to the abundant literature on tablets in general (Behera et al., 2003; Siu et al., 2005; Rautio and Keski-Nisula, 2006; Partanen et al., 2009; Lindfors et al., 2010).

(Devulapalli et al., 2015) published a review about intra-arterial injections (all molecules combined) of the upper limb, gathering 209 patients from 25 articles. The mean age was 31 years with a majority of men. The most common injection site was the brachial artery, and the most commonly used substances were opiates on prescription (25%). It is interesting to note that the key time limit to reduce the risk of amputation was 14 h in case of injection of pure substances (i.e. heroin, cocain). Of the 52 patients presenting over 14 h after the intra-arterial injection, 46% required amputation whereas only 4% of the 50 patients presenting within 14 h. This may explain the different outcomes of the case from (Boucher et al., 2008) who presented within 4 h, and was amputated of only one phalanx, and the case from (Chang and Lin, 2003) or our case, who presented 48 and 72 h respectively after the intra-arterial injection, and for whom more extensive amputations were required. However, in case of injection of crushed tablets whatever the active substance and time interval, the incidence of amputation was 6 times higher than in case of injection of pure substances in (Devulapalli et al., 2015). Indeed, injection of solid material corresponding to crushed tablets may lead to a greater loss of arterial bed. For this reason, injections of solid material might be considered as more severe, requiring therefore a shorter, although not precisely determined, rescue time. The patient we report here, rapidly presented to the emergency room for pain, probably within 14 h after the intra-arterial injection. But she did not mention it, and the diagnosis was not made. When she came to our hospital 72 h later, she had exceeded the delay to avoid any amputation.

In terms of therapeutic management, 53% of the patients in (Devulapalli et al., 2015) review received antiplatelet therapy and 77% an anticoagulant. One quarter received vasodilators, including iloprost. The analysis showed that anticoagulation did not confer any advantage on the amputation rate. Only corticosteroids seemed to be associated with a lower amputation rate, but their prescription in intra-arterial obstructions remains cautious given the lack of knowledge on their effects in this context, in contrast with the increased risk of infection. (Devulapalli et al., 2015) also pointed out that the type of substance injected carried out different risks of amputation, with benzodiazepines in the lead with a 50% incidence.

We tried to find out some mechanistic explanations to the increased risk of necrosis after benzodiazepine intra-arterial injection and whether these hypotheses could be shared with zolpidem, a benzodiazepine-like imidazopyridine derivative. The higher ischemic risk of this related benzodiazepines may be predominantly due to their insolubility, rather than their pharmacologic effects on the arterial wall. We found out that all the manufacturers incorporate microcrystalline cellulose into the core of zolpidem tablets. A review of the literature let out several cases with severe ischemia after intra-arterial injections with drugs having microcrystalline cellulose among their components: methadone (Gramenz et al., 2010) meprobamate (Seak et al., 2012) and codeine tablets (Goldberg et al., 1984). Microcrystalline cellulose is a primary excipient in pharmaceutical oral formulations because of its very low reactivity to active ingredients. It is insoluble in water and most organic solvents, and resistant to degradation in water and is water-absorbent, which result in reduced particle agglomeration. They are fundamentally globoid in shape and come in different diameters (150–500 µm). They are uniform and accurately calibrated size (Kai et al., 2000). Their small diameter explains that the cases recorded so far concern ischemia and digital necrosis, the microsphere totally occluding the artery of very small caliber of interest, which is confirmed in our case report. The beads migrated distally, thereby producing a complete and permanent occlusion. Their tendency to travel to vessels with diameters approximating their own resulted in a high embolic effect (Kai et al., 2000). (Goldberg et al., 1984) tested each of the components of the tablet the patient had self-injected. Codeine, lactose, gelatin, carboxymethyl cellulose, calcium stearate, talc, and microcrystalline cellulose were separately injected into the femoral arteries of dogs. The unique component producing the gangrene was the microcrystalline cellulose, while the injection of pure codeine was harmless.

Lastly, we checked out scientific works focusing on two benzodiazepine-related mechanisms: first, on their inhibition properties on phosphodiesterase, a vasodilator intermediate, and second on the peripheral benzodiazepine-type receptor (PBR), which could take part in response to ischemia. Most of works on the inhibition of the phosphodiesterase have been done in cardiology and with diazepam and its positive inotropic effect (Carmen Collado et al., 1998). But no impact on phosphodiesterase type 1, the most involved in peripheral vasodilation and no data on arterial vasoconstriction with diazepam were reported (Chan and Yan, 2011). We did not find similar effects with zolpidem. PBR expression level was also found to be increased in the arterial plaque of patients with atherosclerosis and inflammation (Veenman and Gavish, 2006), suggesting that a high expression of PBR is strongly correlated with tissue inflammation. Moreover, PBR in the arterial wall appear to take part in the response to ischemia (Joo et al., 2012). But zolpidem does not seem to be a ligand of PBR (Berson et al. 2001), which refutes this hypothesis.

Thus, we believe that the ischemic process observed in our patient is more likely in relation with the microcrystalline cellulose component and its insolubility rather than with zolpidem and its pharmacology effects.

As a conclusion, in patients looking for venous access, injections of pure substances or crushed tablets end most often unintentionally in an artery. Every physician should be aware of the danger of accidental intra-arterial injections, pay attention to any sign of drug abuse in case of acute upper extremity ischemia or pain, in order to quickly start antithrombotic therapy with vasodilators. Medical management is still debated and not consensual. However, anticoagulation does not seem to be mandatory over antiplatelet therapy. Main predictive factors of amputation are the delay to presentation after the intra-arterial injection, and the type and form of substance injected. One should not forget to take into account the excipients associated with the drug injected, in particular in case of crushed tablets. These factors may influence the functional prognosis of the hand in case of microcrystalline cellulose injection.

## Take-Away Lessons From the Case

• There is a critical threshold of 14 h in the management of acute digital ischemia to limit the risk of amputation.
• The use of anticoagulants does not seem to decrease the amputation rate, which would be more related to the type of substance injected.
• Microcrystalline cellulose, one of the excipients of zolpidem, are most likely the cause of acute ischemia with this drug after intra-arterial injection.
• Physician should pay attention to any sign of drug abuse in case of acute upper extremity ischemia or pain, and collect the time from injection, the type of drug, and the different components of the tablet crushed, especially microcrystalline cellulose.

## Data Availability Statement

The raw data supporting the conclusions of this article will be made available by the authors, without undue reservation.

## Ethics Statement

Ethical review and approval was not required for the study on human participants in accordance with the local legislation and institutional requirements. The patients/participants provided their written informed consent to participate in this study. Written informed consent was obtained from the individual for the publication of any potentially identifiable images or data included in this article.

## Author Contributions

GD, AG, and TM conceived and wrote the research project, and wrote the manuscript. GG, LK, MG, AL-LL, and EM performed literature review, edited and proofread the manuscript. All authors read and approved the final manuscript.

## Conflict of Interest

The authors declare that the research was conducted in the absence of any commercial or financial relationships that could be construed as a potential conflict of interest.
